# Association between dietary niacin intake and cognitive function in the elderly: Evidence from NHANES 2011–2014

**DOI:** 10.1002/fsn3.3428

**Published:** 2023-05-16

**Authors:** Xia Shen, Long Yang, Yuan Yuan Liu, Lei Jiang, Jian Feng Huang

**Affiliations:** ^1^ Department of Nursing Affiliated Hospital of Jiangnan University Wuxi China; ^2^ Department of Nursing, Wuxi Medical College Jiangnan University Wuxi China; ^3^ College of Pediatrics Xinjiang Medical University Urumqi China; ^4^ Department of Radiology The Convalescent Hospital of East China Wuxi China; ^5^ Department of Radiation Oncology Affiliated Hospital of Jiangnan University Wuxi China

**Keywords:** cognitive function, cognitive impairment, cross‐sectional, dose–response, elderly, niacin

## Abstract

Recent studies have shown an inconsistent association between dietary niacin and cognitive function. And this remains unclear in the American outpatient population. The aim of this study was to assess whether there is an association between dietary niacin and cognitive performance in an older American population aged ≥60 years. A total of 2523 participants from the National Health and Nutrition Examination Survey (NHANES) 2011–2014 were enrolled. Cognitive function was assessed by the CERAD Word Learning (CERAD‐WL) test, the CERAD Delayed Recall (CERAD‐DR) test, the Animal Fluency test (AFT), and the Digit Symbol Substitution test (DSST). Cognitive impairment that meets one of the four scoring conditions listed above is defined as low cognitive function. Dietary niacin intake was obtained from 2 days of a 24‐h recall questionnaire. Based on the quartiles of dietary niacin intake, they were divided into four groups: Q1 (<15.51 mg), Q2 (15.51–20.68 mg), Q3 (20.69–26.90 mg), and Q4 (>26.91 mg). The stability of the results was assessed using multifactorial logistic regression, restricted cubic spline (RCS) models, and sensitivity stratified analysis. More than half of the participants had cognitive impairment (52.52%). In the fully adjusted model, niacin was associated with a significantly reduced risk of cognitive impairment in Q3 and Q4 compared with the Q1 group (OR: 0.610, 95% CI: 0.403, 0.921, *p* = .022; OR: 0.592, 95% CI: 0.367, 0.954, *p* = .034). Meanwhile, niacin was negatively associated with poor cognition as assessed by the CERAD‐WL test, CERAD test, AFT, and DSST. An L‐shaped dose–response relationship between dietary niacin and cognitive function was observed in all participants (nonlinear *p* < .001). There were also interactions that existed in populations with different carbohydrate intakes and cholesterol intakes (*p* for interaction = .031, *p* for interaction = .005). These findings provide new evidence for the potential role of dietary niacin intake on cognitive function in the elderly.

## Graphical abstract

We derived a negative association between dietary niacin and low cognition. Our study may be able to provide some reference for dietary prevention and treatment strategies for people with cognitive hypoacusis, which has significant implications for the management of patients with impaired cognitive function.

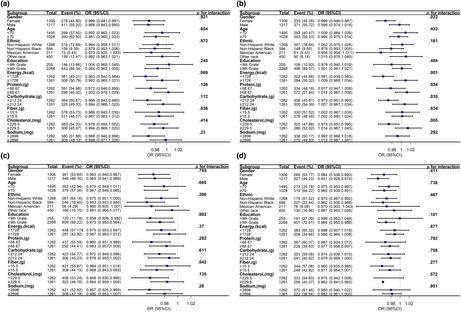

## 1 INTRODUCTION

With the increase in human life expectancy, the cognitive health of older adults has become one of the major global public health issues (Ostojic et al., 2021). The number of older adults with cognitive impairment or dementia is growing rapidly, and it is estimated that this population will reach 65.7 million by 2030, increasing to 115.4 million by 2050 (Afzal et al., 2014; Prince et al., 2013). Impaired cognitive function refers to impairments in a variety of mental abilities, including poor concentration, memory loss, information processing and remembering confusion or loss, semantic comprehension errors, and abnormal reasoning and decision‐making (Morozova et al., 2022). Cognitive decline can seriously affect the activities of daily living of older adults and reduce their quality of life (Lai et al., 2019). Cognitive decline is a prominent feature of dementia, which is the fifth leading cause of death among the elderly and imposes a heavy medical and psychological burden on patients and families (Weng et al., 2022; Wimo et al., 2013). The course of dementia is progressive and irreversible, and to date, there is no effective treatment for dementia. Therefore, it is crucial to explore preventive strategies that may be effective against cognitive impairment.

Some studies have reported that cognitive decline appears to be inextricably linked to physical, psychological, social, and lifestyle factors as well as dietary factors (Khalsa, 2015; Rosenberg et al., 2020; Wang et al., 2021; Zhang et al., 2022). Dietary factors, as one of the potential influencing contributors, have been abundantly shown to be strongly associated with brain volume loss or brain integrity (Raji et al., 2014; Stomby et al., 2017). A previous review reported that folic acid, flavonoids, vitamin D, and certain lipids or foods (such as fruits, vegetables, fish, and whole grains) have a protective effect on cognition in older adults (Scarmeas et al., 2018). Several recent studies have shown that dietary fiber, caffeine, B vitamins, and vitamin C are strongly associated with cognitive function (Aisen et al., 2008; Dong et al., 2020; Prokopidis et al., 2022; Travica et al., 2017).

Niacin, a key vitamin, one of the B vitamins, is present as nicotinamide (pyridine‐3‐carboxamide) and nicotinic acid (pyridine‐3‐carboxylic acid) (D'Andrea et al., 2019). It is a precursor of nicotinamide adenine dinucleotide and nicotinamide adenine dinucleotide phosphate (Afzal et al., 2017). Niacin is widely found in animal and plant products, such as fortified cereals, meats, or vegetables (Mielgo‐Ayuso et al., 2018). Deficiency in niacin and tryptophan is known to cause pellagra, which is characterized by dementia, diarrhea, and dermatitis. Thus, a biological link between niacin and cognition seems plausible. In addition, niacin is also beneficial in the prevention and treatment of aging, cancer, and metabolic diseases (Wang et al., 2022). A clinical study suggests that niacin supplementation has beneficial effects on cognitive function in later life (Ma et al., 2016). Furthermore, several studies (Gasperi et al., 2019; Moutinho et al., 2022; Pirinen et al., 2020) have shown that adequate niacin plays an essential role in DNA synthesis and repair, myelination and dendritic growth, cellular calcium signaling, and as a potent antioxidant in brain mitochondria, which are associated with proper brain function.

To our knowledge, there are few and inconsistent studies examining the association between dietary niacin intake and cognitive impairment in the populations (Morris et al., 2004; Qin et al., 2017; Sheng et al., 2020). Moreover, the relationship is unclear in outpatient geriatric patients in the United States. We aimed to explore the relationship between dietary niacin and cognitive function using data from the National Health and Nutrition Examination Survey (NHANES). This study was specific to dietary niacin, and to make our findings more representative and accurate, so much so that niacin supplementation was included as a covariate to eliminate its potential impact on the results. We hypothesized that dietary niacin intake would be low in patients with cognitive impairment.

## 2 MATERIALS AND METHODS

### 2.1 Study population

**FIGURE 1:**
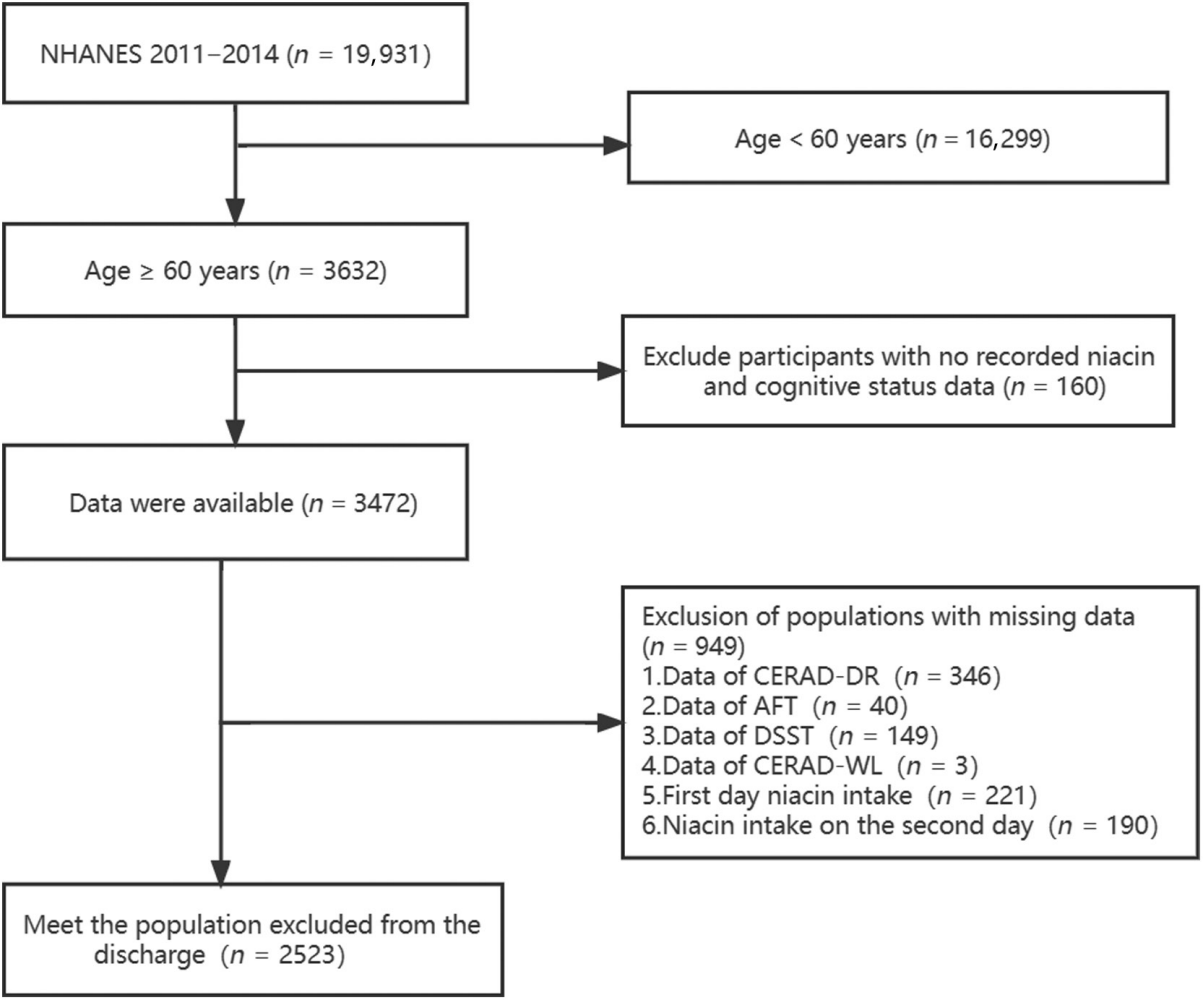
The flow chart of inclusion and exclusion criteria in the study.

Data from two consecutive National Health and Nutrition Examination Survey (NHANES) cycles from 2011 to 2014. The NHANES database is based on a stratified, multistage, and probability cluster designed and is mobilized by the National Center of Health Statistics of the Centers for Disease Control and Prevention (Huang et al., 2021; Sahakyan et al., 2015). A mobile examination center (MEC) was used to perform physical examinations and collect blood samples. It includes demographic data, dietary interviews, laboratory tests, and examinations performed by professionally trained staff (Prevention CfDCa. NHANES Questionnaires). In this study, we selected a population with greater than or equal to 60 (*n* = 3632), excluding the participants with no records of niacin intake and cognitive status (160) and deleting the data for missing cognitive examination (538) and niacin intake (411), leaving a final sample of 2523. Additional details of the study sampling and exclusion criteria are shown in Figure 1. Data were analyzed from November 2022 to January 2023. This study strictly followed the Strengthening the Reporting of Observational Studies in Epidemiology (STROBE) (Vandenbroucke et al., 2007). Furthermore, this study was supported by the National Center for Health Statistics Research Ethics Review Board, and the ethics approval numbers are Protocol #2011–17 and Continuation of Protocol #2011‐17. You can find it at this website: NCHS Ethics Review Board Approval (cdc.gov). And all the data used in the manuscript can be available on the website: https://wwwn.cdc.gov/nchs/nhanes/search/default.aspx. Everyone provided informed consent.

### 2.2 Diagnosis of cognitive impairment assessment

Four tests were used to assess cognitive function (CDC. National Health and Nutrition Examination Survey 2011–2012 Data Documentation) in participants aged 60 years or older: including the Delayed Recall Test (DRT), the Immediate Recall Test (IRT), the Animal Mobility Test (AFT), and the Digit Symbol Substitution Test (DSST). These tests have been used in epidemiological, large screening, and clinical studies to assess memory, language, executive function, and processing speed (Fillenbaum et al., 2008; Jaeger, 2018).

The IRT and DRT belong to the Consortium to Establish a Registry for Alzheimer's Disease‐Word Learning (CERAD‐WL) test and Consortium to Establish a Registry for Alzheimer's Disease‐Delayed Recall (CERAD‐DR) test, which are used to assess immediate and delayed learning of new verbal information (memory subdomains). The CERAD‐WL test has three consecutive tests CERAD‐WL1, CERAD‐WL2, and CERAD‐WL3. Participants were asked to read aloud 10 unrelated words, one at a time, and the order of the 10 words had to be changed for each learning trial. We averaged the three CERAD‐WL test scores to obtain the final score, which was determined to be cognitively impaired when it was less than 17. In contrast, on the CERAD‐DR test, participants had to recall the 10 unrelated words used in the first CERAD‐WL test after all cognitive performance tests were completed, a time approximately 8–10 min from the beginning of the word learning trial. Scores greater than 4 on the CERAD‐DR test were considered cognitively normal, otherwise, they were cognitively impaired. Participants on the DSST were asked to replicate the corresponding symbols in 133 boxes within 2 min, with scores greater than 33 considered cognitively normal. The AFT (Canning et al., 2004) was used to test verbal fluency, and participants were asked to recall as many animals as possible within 1 min. When the AFT score was less than 14, participants were considered to have a cognitive decline. In addition, we defined cognitive impairment as meeting any of the four tests described above. For more information on the scores, please refer to the 1999–2000 NHANES CFQ questionnaire data file, which can be accessed at this URL: https://wwwn.cdc.gov/Nchs/Nhanes/1999‐2000/CFQ.htm.

### 2.3 Niacin intake

Dietary intake data are collected using the NHANES Computer‐Aided Dietary Interview (CADI) system by trained dietary interviewers who are fluent in Spanish and English, and this fully computerized recall system is comprehensively composed of standard food‐specific questions and possible answers. Each MEC dietary interview room has a standard set of measurement guidelines. These tools can be used to help respondents report the volume and size of food consumed. Moreover, this set of measurement guidelines was specifically designed for use in the current NHANES setting and was agreed upon by experts during regular workshops to evaluate NHANES data collection (Ahluwalia et al., 2016). The NHANES Dietary Interviewer Procedures Manual involves a complete overview of dietary survey methods (Liu et al., 2022). To make the results more convincing, we used the average of 2 days of dietary niacin intake in the study. And niacin in this study refers to dietary niacin and does not include supplemental niacin. The categorical variables of niacin were defined according to the participants' dietary niacin intake and divided into four groups.

### 2.4 Covariate assessments

The selection of covariates was based on clinical experience and previous literature (Jung et al., 2018; Li et al., 2019; Prokopidis et al., 2022). Age, gender, race/ethnicity, marital status, education levels, alcohol consumption, and smoking status were obtained from in‐person household interviews. Race/ethnicity was categorized as non‐Hispanic white, non‐Hispanic black, Mexican American, and other. Education levels were classified as less than ninth grade, and higher than ninth grade, or ninth grade. Marital status was categorized as married (married, living with a partner) and not married (widowed, divorced, separated, or never married). Participants were categorized as “mild”, “heavy”, and “no” based on the number of drinks per day he/she had drunk in 1 year. Participants who are “mild” were considered to be drinking alcohol ≤1 drink in women and ≤2 drinks in men; Participants who are “heavy” were considered to be drinking alcohol ≤2 drinks in women and ≤ 3 drinks in men or individuals had drunk ≥3 drinks in woman and ≥ 4 drinks in man; Those who drank before but do not drink now and those who never drank before are defined as “no” (Rattan et al., 2022). Smoking status was defined as the number and timeline of cigarettes in life (no, smoked less than 100 cigarettes or smoked more than 100 cigarettes in life and smoke not at all now; yes, smoked ≥100 cigarettes in life and smoke some days or every day). BMI (body mass index) equals weight (kg) divided by height (m) squared (Shachar et al., 2017). Physical activity is calculated based on the amount of exercise per day multiplied by the MET (metabolic equivalent task) of exercise intensity (Lee et al., 2016). When an individual's MET for physical activity is <600 min/week, he is considered as low‐intensity physical activity; when an individual's MET for physical activity is >8000 min/week, he is considered as high‐intensity physical activity; when the MET is in between 600 and 7999 min/week, he is considered as medium intensity physical activity (Fan et al., 2022). Trained staff used a 24‐h recall method to determine dietary intake, including protein, carbohydrates, cholesterol, fiber, vitamin B1, vitamin B2, sodium, niacin, and niacin supplements. In selecting the diet data, we used the average of the first and the second day's interview data for estimation. Blood indicators such as albumin, total cholesterol, and triglyceride are taken from the Laboratory Data section. Patients were asked if they were taking any medications. Patients not taking niacin and those taking other medications were defined as “no”, and those taking niacin were defined as “yes”. Diabetes was defined as a diagnosis from a doctor or other health professional, 2‐h OGTT blood glucose (mmol/l) ≥ 11.1, random blood glucose (mmol/L) ≥ 11.1, fasting glucose (mmol/l) ≥ 7.0, HbA1c (%) >6.5, or use of medication or insulin. CVD consisted of coronary heart disease or heart attack or stroke and was assessed by asking participants about their diagnoses. Participants had three blood pressure measurements performed, and the mean of the three measurements was defined as systolic blood pressure (SBP) and diastolic blood pressure (DBP). Hypertension was defined as a self‐reported history of hypertension or use of antihypertensive medication or SBP ≥140 mmHg or DPB ≥90 mmHg.

### 2.5 Statistical analysis

We considered complex sampling designs and sample sizes during data analysis (Ruan et al., 2022) according to NHANES analysis guidelines. And the present data can represent a sample population of 55,245,547. Continuous variables were expressed as weighted mean ± standard deviation, and one‐way ANOVA was used to compare differences between groups. Categorical variables were expressed as frequencies and percentages and compared using Rao Scott's χ^2^ test. A two‐sided *p*‐value less than .05 indicate a denoted statistically significant difference. All analyses were performed using the statistical software package R (http://www.r‐project.org; version 4.2.2, The R Foundation).

The logistic regression model was used to calculate the odds ratios (OR) and 95% confidence interval (CI) for the relationship between niacin and the prevalence of cognitive impairment, and the categorical Q1 group of niacin (< 15.51 mg) was used as a reference. For the selection of variables used for adjustment, we selected statistically significant variables and combined them with clinical as well as covariates of covariance. We used both the unadjusted and adjusted models in the data analysis process. In Model 1, we adjusted for age, gender, ethnicity, marital status, income, educational levels, and alcohol use. We further adjusted for SBP, niacin supplement, drug of niacin, albumin, total cholesterol, and the covariates of Model 1 (Model 2). Furthermore, restricted cubic spline (RCS) regressions were performed at the 5th, 35th, 65th, and 95th percentiles of dietary niacin intake nodes to assess nonlinearity and validate the dose–response relationship between dietary niacin intake and cognitive function after adjusting for all variables in Model 2. Moreover, we performed a corresponding sensitivity stratification analysis to investigate the relationship between dietary niacin and cognitive function in populations with relevant covariate dietary variables. In addition, we also examined the relationship between low cognition and dietary niacin in the CERAD‐DR test, CERAD‐WL test, AFT, and DSST test scores, respectively.

## 3 RESULTS

### 3.1 Participant characteristics according to dietary niacin intake quartiles

In this study, we selected two continuous NHANES cycles (2011–2012 and 2013–2014) and focused on 2523 participants with completed interviews and MEC examination in the US (≥ 60 years). The baseline characteristics of the participants are summarized in Table 1 according to dietary niacin intake quartiles. Among the 2523 participants in the study, there were 51.76% males and 48.24% females recruited and 1325 (52.52%) of the population suffered from cognitive abnormalities. Based on the weighted analyses, the mean age of the 2523 participants was 69.09 years (range, 68.84–69.34 years) and those with an education of above ninth grade accounted for 89.89%, and most of the participants were non‐Hispanic white (50.26%). Participants with higher niacin intake were more likely to be male, Mexican American, married, mild‐physical active individuals, no‐smoking, drinkers, hypertensive, and higher dietary consumers of niacin supplement, energy, protein, carbohydrate, cholesterol, vitamin B1, vitamin B2, and sodium. BMI, smoking status, physical activity, TG, DBP, CVD, diabetes, and hypertension did not differ in the quartile of dietary niacin intake.

**TABLE 1:** Basic information of the population grouped according to niacin.

Characteristics	Total	Q1 (<15.51)	Q2 (15.51–20.68)	Q3 (20.69–26.90)	Q4 (>26.91)	*p*‐value
	2523	631	632	629	631	
Niacin (mg)	23.29 ± 0.35	12.33 ± 0.14	18.11 ± 0.10	23.54 ± 0.11	34.94 ± 0.47	<.001
Age (years)	69.09 ± 0.25	70.13 ± 0.35	69.35 ± 0.38	69.07 ± 0.45	68.17 ± 0.37	.002
Gender (%)						
Female	1306 (51.76)	457 (81.43)	361 (65.33)	304 (50.03)	184 (26.73)	<.001
Male	1217 (48.24)	173 (18.57)	270 (34.67)	327 (49.97)	447 (73.27)	
BMI (kg/m^2^)	29.18 ± 0.25	29.43 ± 0.39	29.00 ± 0.45	29.05 ± 0.44	29.27 ± 0.47	.779
Ethnicity (%)						
Non‐Hispanic White	1268 (50.26)	274 (70.61)	315 (79.11)	338 (80.86)	341 (82.66)	.002
Non‐Hispanic Black	594 (23.54)	178 (13.21)	156 (8.80)	131 (7.39)	129 (6.47)	
Mexican American	211 (8.36)	48 (4.33)	53 (3.10)	51 (2.88)	59 (3.50)	
Other race	450 (17.84)	130 (11.85)	107 (9.00)	111 (8.87)	102 (7.37)	
Marital status (%)						
Not married	1045 (41.42)	314 (46.10)	274 (35.27)	235 (30.60)	222 (25.86)	<.001
Married	1478 (58.58)	316 (53.90)	357 (64.73)	396 (69.40)	409 (74.14)	
Income (%)						
≥20,000$	1839 (72.89)	420 (74.82)	442 (78.62)	491 (85.59)	486 (85.93)	.006
<20,000$	684 (27.11)	210 (25.18)	189 (21.38)	140 (14.41)	145 (14.07)	
Education (%)						
Less than 9th grade	255 (10.11)	82 (9.30)	64 (4.59)	59 (5.71)	50 (3.41)	.006
Higher than 9th grade or 9th grade	2268 (89.89)	548 (90.70)	567 (95.41)	572 (94.29)	581 (96.59)	
Alcohol use (%)						
No	1099 (43.56)	329 (49.47)	292 (39.43)	262 (38.40)	216 (24.37)	<.001
Mild	1002 (39.71)	218 (37.61)	246 (43.85)	254 (44.80)	284 (55.81)	
Heavy	422 (16.73)	83 (12.91)	93 (16.72)	115 (16.81)	131 (19.82)	
Smoking (%)						
No	2226 (88.23)	546 (88.62)	563 (91.54)	568 (92.38)	549 (89.79)	.343
Yes	297 (11.77)	84 (11.38)	68 (8.46)	63 (7.62)	82 (10.21)	
Physical activity (%)						
Low	677 (26.83)	175 (25.37)	185 (24.57)	175 (27.33)	142 (21.12)	.121
Mild	1646 (65.24)	403 (67.31)	416 (71.70)	406 (66.14)	421 (69.69)	
High	200 (7.93)	52 (7.32)	30 (3.73)	50 (6.52)	68 (9.18)	
Energy (kcal)	1913.64 ± 20.90	1252.27 ± 22.62	1710.25 ± 36.54	1986.54 ± 27.46	2470.06 ± 36.51	<.001
Protein (g)	74.97 ± 0.89	45.75 ± 0.85	63.01 ± 0.82	78.86 ± 1.04	101.36 ± 1.27	<.001
Carbohydrate (g)	231.65 ± 3.24	159.32 ± 2.23	209.17 ± 6.28	239.29 ± 5.11	293.00 ± 4.97	<.001
Fiber (g)	17.53 ± 0.31	12.54 ± 0.33	15.78 ± 0.41	17.43 ± 0.44	22.53 ± 0.61	<.001
Cholesterol (mg)	268.11 ± 3.85	179.30 ± 7.73	235.12 ± 7.15	289.88 ± 7.88	336.02 ± 8.30	<.001
Vitamin B1 (mg)	1.54 ± 0.02	0.94 ± 0.02	1.26 ± 0.03	1.59 ± 0.02	2.13 ± 0.04	<.001
Vitamin B2 (mg)	2.10 ± 0.03	1.32 ± 0.04	1.74 ± 0.04	2.15 ± 0.04	2.88 ± 0.07	<.001
Sodium (mg)	3168.36 ± 45.56	2049.05 ± 37.90	2767.99 ± 53.16	3352.16 ± 67.36	4098.39 ± 55.38	<.001
Albumin (g/L)	42.01 ± 0.11	41.44 ± 0.15	42.06 ± 0.20	42.13 ± 0.21	42.26 ± 0.20	.017
TG (mmol/L)	1.39 ± 0.03	1.45 ± 0.06	1.41 ± 0.04	1.37 ± 0.04	1.34 ± 0.04	.197
TC (mmol/L)	4.99 ± 0.03	5.17 ± 0.06	5.06 ± 0.08	4.95 ± 0.07	4.86 ± 0.06	.004
SBP (mmHg)	130.66 ± 0.50	133.54 ± 1.08	128.67 ± 1.16	132.96 ± 0.93	128.09 ± 1.09	<.001
DBP (mmHg)	68.47 ± 0.41	68.48 ± 0.64	67.52 ± 0.61	69.06 ± 0.81	68.67 ± 0.78	.322
CVD (%)						
No	1959 (77.65)	483 (77.23)	487 (76.65)	501 (80.37)	488 (77.76)	.547
Yes	564 (22.35)	147 (22.77)	144 (23.35)	130 (19.63)	143 (22.24)	
Diabetes (%)						
No	1431 (56.72)	333 (58.98)	359 (65.64)	356 (63.77)	383 (64.71)	.506
Yes	1092 (43.28)	297 (41.02)	272 (34.36)	275 (36.23)	248 (35.29)	
Hypertension (%)						
No	738 (29.25)	153 (27.62)	192 (36.01)	176 (30.31)	217 (36.86)	.143
Yes	1785 (70.75)	477 (72.38)	439 (63.99)	455 (69.69)	414 (63.14)	
Niacin supplement (%)						
No	1683 (66.71)	448 (67.83)	437 (66.51)	407 (56.58)	391 (52.41)	<.001
Yes	840 (33.29)	182 (32.17)	194 (33.49)	224 (43.42)	240 (47.59)	
Niacin drugs (%)						
No	2506 (99.33)	627 (99.77)	629 (99.90)	625 (98.62)	625 (98.78)	.063
Yes	17 (0.67)	3 (0.23)	2 (0.10)	6 (1.38)	6 (1.22)	
Cognitive (%)						
No	1198 (47.48)	263 (50.35)	281 (57.57)	324 (61.60)	330 (63.43)	.028
Yes	1325 (52.52)	367 (49.65)	350 (42.43)	307 (38.40)	301 (36.57)	

*Note*: Continuous variables were expressed as weighted mean ± standard deviation, one‐way ANOVA was used to compare differences among the different groups. Categorical variables were expressed as frequencies and percentages and compared using Rao‐Scott's χ2 test.

Abbreviations: BMI, body mass index; CVD, severe cardiovascular diseases; DBP, diastolic blood pressure; SBP, systolic blood pressure; TC, total cholesterol; TG, triglyceride.

### 3.2 Association between dietary niacin intake and cognitive function

Table 2 demonstrates the relationship between dietary niacin intake and cognitive tests in a multifactorial regression, after full adjustment for age, gender, ethnicity, marital status, income, educational levels, alcohol use, SBP, niacin supplement, drug of niacin, albumin, and total cholesterol. We found a significant negative association between dietary niacin intake and low cognition when using quartiles to analyze dietary niacin intake. High niacin intake favors a decrease in the incidence of low cognition. Compared with lower niacin intake (Q1: < 15.51 mg), the weight OR values between dietary niacin and low cognition in Q2 (15.51–20.68 mg), Q3 (20.69–26.90 mg) and Q4 (> 26.91 mg) were 0.855 (95% CI: 0.619–1.181; *p* = .317), 0.610 (95% CI: 0.403, 0.921, *p* = .022), and 0.592 (95% CI: 0.367, 0.954, *p* = .034), respectively. Meanwhile, when niacin intake was greater than 20.69 mg, similar results as described above were observed between low cognition and niacin as demonstrated in the CERAD‐WL test, AFT, and DSST. Besides, Table 2 shows that niacin and low cognition were statistically significant only when niacin intake was between 20.69 and 26.90 mg in the CERAD‐DR test (OR: 0.645, 95%CI:0.438, 0.949, *p* = .029).

**TABLE 2:** Weight Multivariate regression analysis between cognitive function and niacin.

Variable	*N* (%)	Crude model		Model 1		Model 2	
		OR 95% CI	*p*‐value	OR 95% CI	*p*‐value	OR 95% CI	*p*‐value
**Cognitive**							
Dietary Niacin (mg)							
Q1	367 (49.55)	Reference		Reference		Reference	
Q2	350 (42.31)	0.748 (0.518,1.079)	.116	0.804 (0.595,1.086)	.146	0.855 (0.619, 1.181)	.317
Q3	307 (38.55)	0.632 (0.484,0.826)	.001	0.615 (0.413,0.914)	.019	0.610 (0.403, 0.921)	.022
Q4	301 (36.57)	0.585 (0.437,0.783)	<.001	0.566 (0.356,0.899)	.019	0.592 (0.367, 0.954)	.034
*p* for trend			.002		.029		.035
**CERAD‐WL Test**							
Dietary Niacin (mg)							
Q1	195 (28.36)	Reference		Reference		Reference	
Q2	186 (22.94)	0.753 (0.504,1.123)	.157	0.741 (0.514,1.069)	.103	0.766 (0.522,1.125)	.16
Q3	158 (20.21)	0.635 (0.482,0.836)	.002	0.582 (0.415,0.815)	.003	0.581 (0.405,0.832)	.006
Q4	150 (17.21)	0.524 (0.383,0.716)	<.001	0.455 (0.293,0.707)	.001	0.467 (0.294,0.741)	.003
*p* for trend			<.001		.003		.005
**CERAD‐DR Test**							
Dietary Niacin (mg)							
Q1	171 (24.47)	Reference		Reference		Reference	
Q2	160 (18.74)	0.713 (0.549,0.924)	.012	0.698 (0.504,0.967)	.032	0.733 (0.514, 1.044)	.081
Q3	141 (18.66)	0.703 (0.529,0.935)	.017	0.647 (0.451,0.928)	.021	0.645 (0.438, 0.949)	.029
Q4	134 (18.66)	0.706 (0.497,1.003)	.052	0.635 (0.403,1.002)	.051	0.656 (0.413, 1.043)	.071
*p* for trend			.093		.068		.079
**AFT**							
Dietary Niacin (mg)							
Q1	226 (29.70)	Reference		Reference		Reference	
Q2	209 (24.27)	0.760 (0.547,1.054)	.097	0.873 (0.619,1.230)	.416	0.936 (0.645, 1.357)	.708
Q3	156 (18.39)	0.529 (0.393,0.713)	<.001	0.607 (0.415,0.886)	.012	0.602 (0.399, 0.909)	.019
Q4	138 (15.85)	0.445 (0.293,0.675)	<.001	0.553 (0.346,0.882)	.016	0.577 (0.350, 0.951)	.033
*p* for trend			<.001		.008		.014
DSST							
Dietary Niacin (mg)							.015
Q1	203 (22.40)	Reference		Reference		Reference	
Q2	156 (13.01)	0.519 (0.376,0.716)	<.001	0.606 (0.396,0.927)	.023	0.648 (0.417, 1.005)	.052
Q3	125 (11.27)	0.437 (0.280,0.682)	<.001	0.514 (0.284,0.932)	.03	0.520 (0.284, 0.953)	.036
Q4	108 (8.61)	0.326 (0.227,0.682)	<.001	0.480 (0.287,0.803)	.008	0.505 (0.294,0.868)	.017
*p* for trend			<.001		.012		.019

*Note*: Calculation using multivariate logistic regression analysis was performed. Crude Model: not adjusted. Model 1: Adjusted for age, gender, ethnicity, marital status, income, education, and alcohol use. Model 2: Adjusted for age, gender, ethnicity, marital status, income, education, alcohol use, SBP, niacin supplement, drug of niacin, albumin, carbohydrate intake, and total cholesterol intake. Q1: <15.51 mg. Q2: 15.51–20.68 mg. Q3: 20.69–26.90 mg. Q4: >26.91 mg.

Abbreviations: AFT, Animal Mobility Test; CERAD‐DR, Establish a Registry for Alzheimer's Disease‐delayed recall; CERAD‐WL, Establish a Registry for Alzheimer's Disease‐Word Learning Test; DSST, Digit Symbol Substitution Test.

### 3.3 Dose–response or nonlinear relationships between cognitive function and dietary niacin intake in older

**FIGURE 2:**
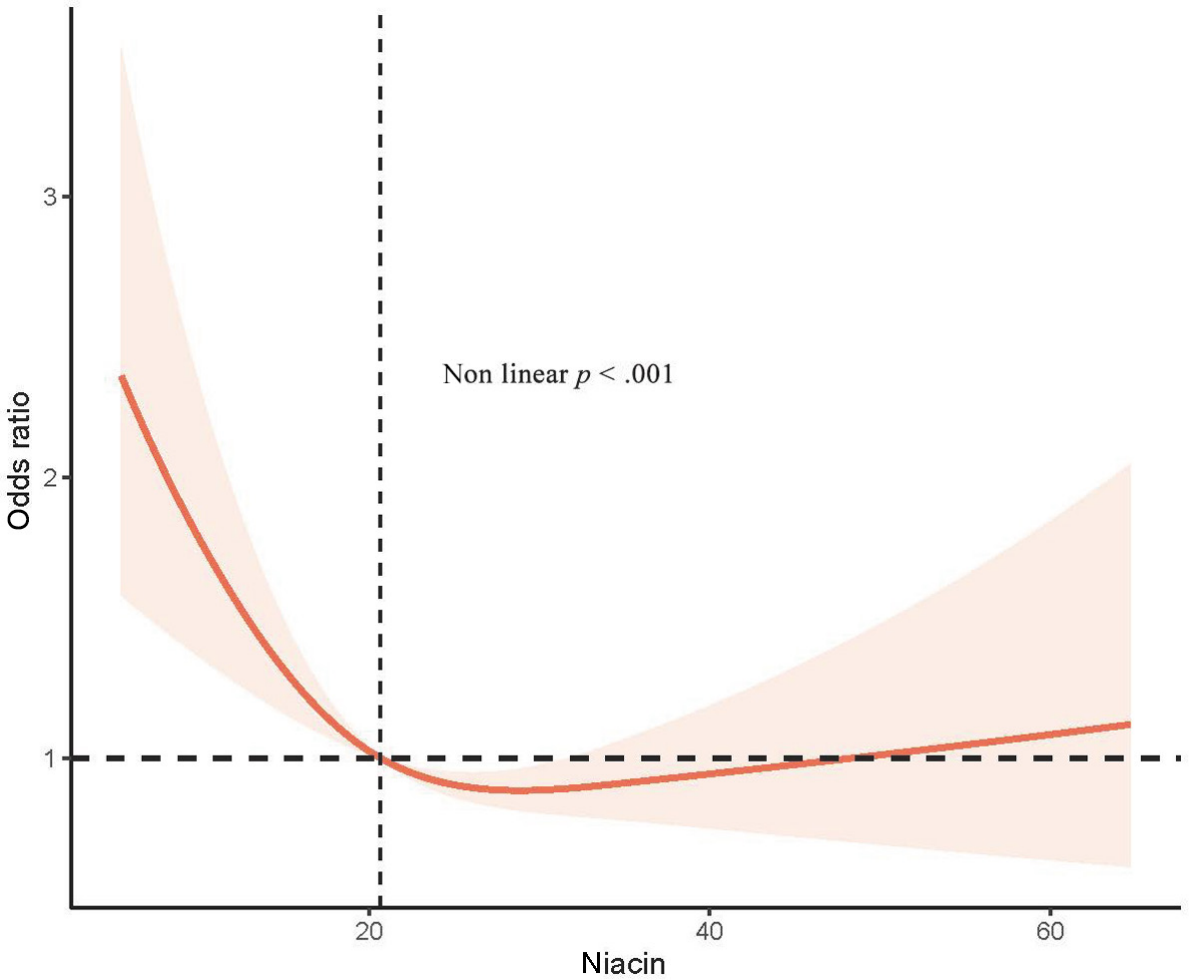
Restricted cubic spline plot of the association between dietary niacin and cognitive function and components. Adjusted for age, gender, ethnicity, marital status, income, education, alcohol use, SBP, niacin supplement, drug of niacin, albumin, carbohydrate intake, and total cholesterol.

**FIGURE 3:**
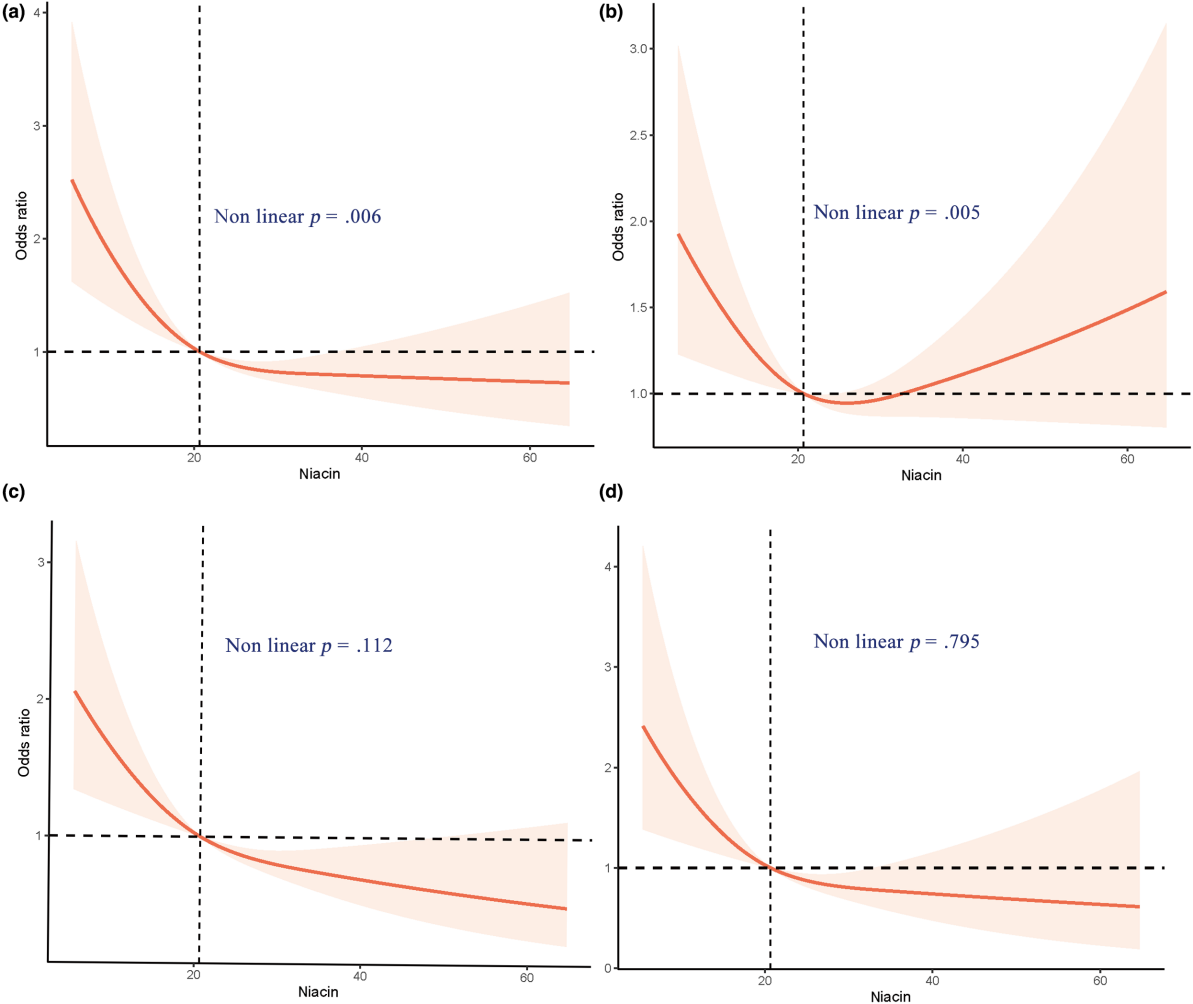
Restricted cubic spline plot of the association between dietary niacin and cognitive function and components. (a) Accessed by CERAD‐WL Test; (b) Accessed by CERAD‐DR Test; (c) Accessed by AFT (Animal Fluency test); (d) Accessed by DSST (Digit Symbol Substitution Test). Adjusted for age, gender, ethnicity, marital status, income, education, alcohol use, SBP, niacin supplement, drug of niacin, albumin, carbohydrate intake, and total cholesterol intake.

In a restricted cubic spline regression analysis model fully adjusted for confounders, we found a linear trend between cognitive function and niacin intake in the AFT and DSST (*p* for trend = .014, *p* for trend = .019) in Table 2 with the dose–response relationship (*p* for nonlinearity = .112) and (*p* for nonlinearity = .795) in (c) and (d) of Figure 3. Figure 2 indicates that cognitive decline determined by testing in any of the four ways showed a nonlinear relationship with niacin (the nonlinearity *p* < .001). However, the dose–response relationship between sustained niacin intake and cognitive impairment showed a general trend of decreasing followed by a slow increase. Moreover, both cognitive depression by the CERAD‐WL test and by CERAD‐DR test showed a nonlinear relationship with dietary niacin (the nonlinearity *p* = .006, the nonlinearity *p* = .005), as shown in A and B in Figure 3.

### 3.4 Subgroup analysis

**FIGURE 4:**
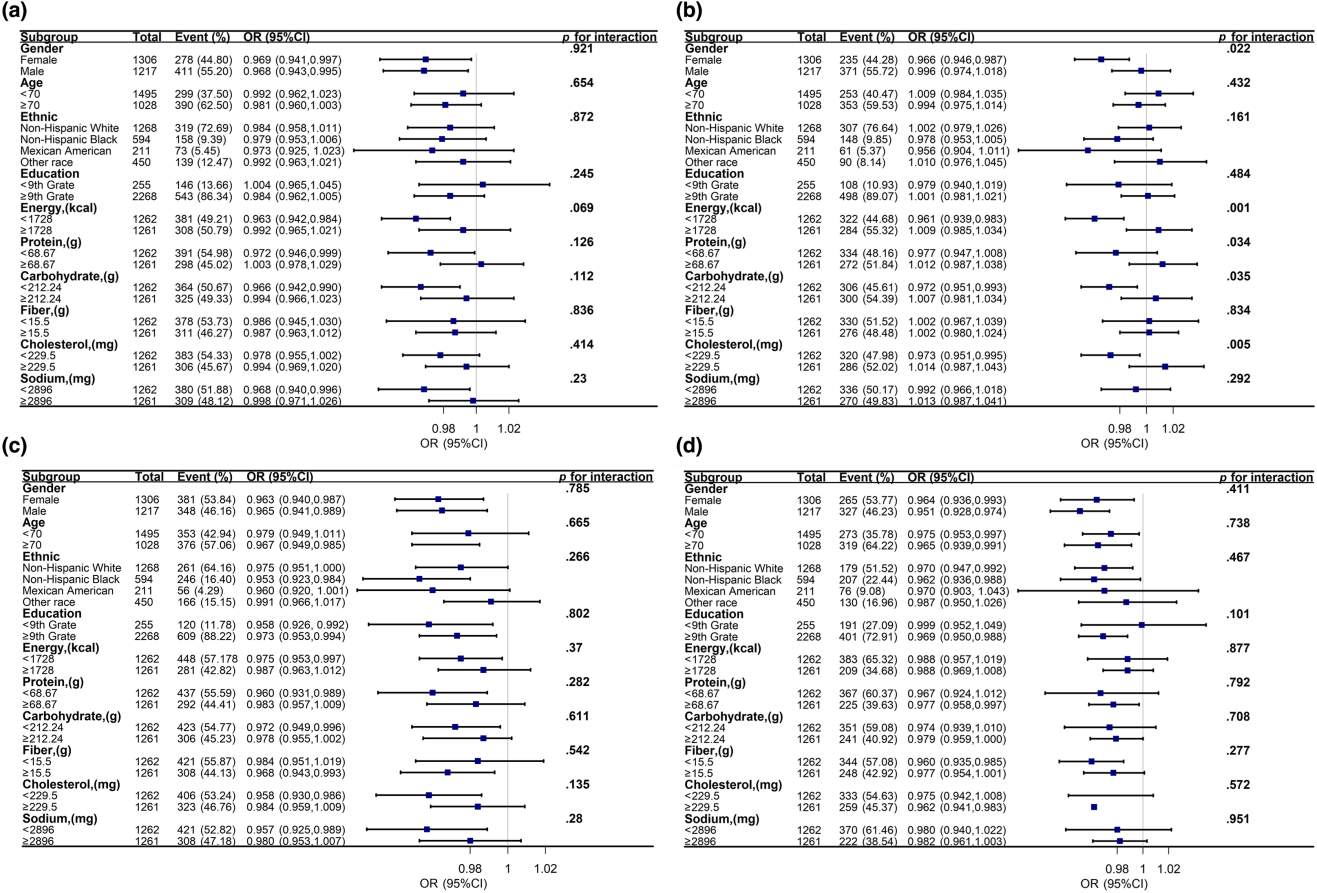
The relationship between dietary niacin and cognitive function in different subgroups of the population. (a) Accessed by CERAD‐WL Test; (b) Accessed by CERAD‐DR Test; (c) Accessed by AFT (Animal Fluency Test); (d) Accessed by DSST (Digit Symbol Substitution Test). Adjusted for marital status, income, alcohol use, SBP, niacin supplement, drug of niacin, albumin, carbohydrate intake, and total cholesterol.

We performed stratified analyses across dietary intake, gender and age, race, and education levels in Table 3. Dietary niacin intake was protective against cognitive depression in subjects who were female, below ninth grade, non‐Hispanic black, with carbohydrate intake <212.24 g and cholesterol intake <229.5 mg, whereas no such relationship was found in other subgroups. Further, Table 3 also found that the relationship between niacin and cognitive function interacted across populations with different carbohydrate intake (*p* for interaction = .031) and cholesterol intake (*p* for interaction = .005). In addition, we investigated the relationship between cognitive impairment and dietary niacin in different subgroups of the population as determined by the CERAD‐DR test, CERAD‐WL test, AFT, and DSST as shown in A, B, C, and D of Figure 4, respectively. There were interactions between cognitive impairment and niacin as determined by the CERAD‐WL test across gender, energy intake, protein intake, carbohydrate intake, and cholesterol intake (*p* for interaction <.05).

**TABLE 3:** Relationship between cognitive function and niacin in different subgroups.

Characteristics	Total	Event (%)	OR (95% CI)	*p*	*p* for interaction
Gender					
Female	1306	609 (47.19)	0.972 (0.949,0.996)	**.011**	.393
Male	1217	716 (52.81)	0.985 (0.960,1.009)	.202	
Age					
<70	1495	657 (45.30)	1.001 (0.977,1.025)	.946	.679
≥71	1028	668 (54.70)	0.994 (0.975,1.014)	.545	
Ethnicity					
Non‐Hispanic White	1268	553 (69.71)	0.995 (0.975,1.016)	.651	.372
Non‐Hispanic Black	594	370 (12.60)	0.969 (0.949,0.990)	**.007**	
Mexican American	211	121 (4.63)	0.972 (0.920,1.027)	.223	
Other race	450	281 (13.11)	0.991 (0.962,1.020)	.519	
Education					
Less than 9th grade	255	230 (11.14)	0.954 (0.919, 0.989)	**.013**	.131
Higher than 9th grade or 9th grade	2268	1095 (88.86)	0.993 (0.975,1.011)	.427	
Energy (kcal)					
<1728	1262	735 (49.49)	0.989 (0.968,1.010)	.287	.133
≥1728	1261	607 (49.89)	1.004 (0.980,1.030)	.717	
Protein (g)					
<68.67	1262	718 (50.11)	0.993 (0.967,1.020)	.582	.392
≥68.67	1261	607 (49.89)	1.004 (0.981,1.027)	.724	
Carbohydrate (g)					
<212.24	1262	695 (49.52)	0.977 (0.956,0.999)	**.043**	**.031**
≥212.24	1261	630 (50.48)	1.006 (0.980,1.032)	.651	
Fiber (g)					
<15.5	1262	714 (52.79)	0.999 (0.971,1.027)	.917	.92
≥15.5	1261	611 (47.21)	1.000 (0.979,1.022)	.977	
Cholesterol (mg)					
<229.5	1262	702 (49.45)	0.977 (0.956,0.998)	**.07**	**.005**
≥229.5	1261	623 (50.55)	1.007 (0.985,1.029)	.53	
Sodium (mg)					
<2896	1262	720 (49.19)	0.984 (0.960,1.009)	.2	.206
≥2896	1261	605 (50.81)	1.004 (0.980,1.029)	.724	

*Note*: Adjusted for marital status, income, alcohol use, SBP, niacin supplement, drug of niacin, albumin, and total cholesterol.

Bold values represent *p* values less than 0.05 and are statistically significant.

## 4 DISCUSSION

In this population‐based study of 2523 American elders aged 60 years and older, we investigated the relationship between dietary niacin intake and cognitive performance. Dietary niacin intake was found to be negatively associated with the risk of low cognitive performance after adjustment for potential confounders. These results are consistent with our hypothesis. Moreover, the correlations between dietary niacin intake and cognitive performance were significantly different across gender, different races, different levels of education, carbohydrate intake, and cholesterol intake stratified analyses. None of these variables significantly altered the nearly L‐shaped relationship between dietary niacin and cognitive dysfunction. Although the *p*‐values for the interactions in these two subgroups were below .05, these results may not be clinically significant given the similar directionality of the multiple tests and associations. Meanwhile, we also found no association between niacin and cognitive function in the dietary subgroups of protein intake, energy intake, fiber intake, and sodium intake, these results further explain why these variables were not included in the model for adjustment in our study. In addition, the probability of low cognitive function found in this study appears to be higher than in several other studies conducted in different countries (Lee et al., 2001; Paulionis et al., 2005; Sheng et al., 2020). These differences may arise due to different definitions of cognitive hyperacusis.

To date, studies have been reported on the relationship between niacin and cognitive function. In support of our research, a large prospective study (Morris et al., 2004) of 6158 residents aged 65 years and older living in the Chicago community from 1993 to 2002 found that higher dietary niacin intake was not only linearly associated with lower cognitive decline (*p* for linear trend = .002), but also with slower annual rates of cognitive decline. Subsequently, a randomized controlled trial (Ma et al., 2016) in China found a beneficial effect of relatively short‐term folic acid supplementation on cognitive function in later life. Furthermore, a community‐based multicenter longitudinal study (Qin et al., 2017) from the United States indicated that a higher intake of niacin‐based foods throughout early adulthood was associated with better cognitive function in midlife. Similarly, a cross‐sectional study from Korea found a significant positive association between cognitive scores and niacin intake (Lee et al., 2001). However, two studies from mixed‐race 65 and older community residents on the south side of Chicago and Chinese Singaporeans found that niacin was not associated with cognitive function (Morris et al., 2006; Sheng et al., 2020). Inconsistencies between studies may be related to differences in statistical methods, adjustment for covariates, and subject characteristics (ethnicity).

To the best of our knowledge, the physiological mechanisms of the association between dietary niacin intake and cognitive decline are not fully understood and may be explained by the following potential pathways. To our knowledge, pathological changes in cognitive impairment are closely associated with the formation of accumulated aggregates containing β‐amyloid, neurofibrillary tangles, and loss of neurons and synapses (Chen, 2018). First, niacin can reduce the inflammatory response to amyloid, enhance phagocytosis, and increase cytokines by improving microglia monitoring (Giri et al., 2019; Rawji et al., 2020). An animal experiment (Moutinho et al., 2022) validated the principle and pathway approach with respect to β‐amyloid. Meanwhile, nicotinic acid has been identified as a ligand for the high‐affinity heterotrimeric guanine nucleotide‐binding protein‐coupled receptor hydroxycarboxylic acid receptor 2 (HCAR2), which regulates cellular autophagy (Wise et al., 2003). HCAR2 in the brain can be selectively expressed by microglia and is strongly induced by amyloid proteins, but the use of Food and Drug Administration (FDA)‐approved niacin preparations in 5xFAD mice can activate HCAR2, thereby reducing plaque load and neuronal dystrophy, ultimately achieving a reduction in neuronal loss and rescuing working memory deficits (Moutinho et al., 2022). Second, B vitamins are known to be involved in homocysteine metabolism, so dietary niacin has the same role as one of the B vitamins. Homocysteine concentrations have been found to be positively correlated with amyloid neurotoxicity (Kruman et al., 2002), increasing the chance of localized brain atrophy, cognitive impairment, or dementia (Douaud et al., 2013; Haan et al., 2007). Third, niacin has been shown not only to contribute to systemic NAD deficiency but also to improve mitochondrial myopathy in adults (Pirinen et al., 2020). The antioxidant stress function of niacin may be related to improving mitochondrial function and promoting NAD cleavage and value addition (Fila et al., 2021), thus supplementing brain energy deficiency to further improve cognitive dysfunction. In addition, niacin affects the release of nitric oxide (Gomaraschi et al., 2015) and tumor necrosis factor α (TNF‐α) (Tavintharan et al., 2007) in the brain, thereby affecting cognitive function. Moreover, brain‐derived neurotrophic factor (BDNF), a pleiotropic secreted protein, plays a key role in the adult brain, where it is closely related to cognition, especially learning and memory‐related structures and functions (Foshati et al., 2022; Miranda et al., 2019). BDNF also activates its receptor tropomyosin‐related kinase B (TrkB), which plays an important role in regulating neuronal migration, differentiation, and control of synaptic function, as well as regulating neuronal survival (Fu et al., 2014). A study (Cui et al., 2010) has reported that niacin administration may upregulate BDNF–TrkB expression. However, the exact mechanism needs to be further investigated.

Our study has several advantages. First, we used a large sample from the MHANES database, so the population size can be guaranteed. Furthermore, we examined the association between dietary niacin and cognitive function, which was determined not only by meeting any one of the four tests but also by each of the four different tests. In addition, we adjusted for many important confounding factors such as niacin medications and niacin supplements, so the results may be more convincing. This study also has several limitations. First, despite our rigorous adjustment for baseline clinical characteristics, our observations may be influenced by unmeasured and unknown confounders. Moreover, the cross‐sectional nature of our study does not allow us to demonstrate causality. Finally, the use of a 24‐h recall questionnaire for dietary intake assessment in NHANES may be somewhat biased, and perhaps a food frequency questionnaire would be a better choice for this type of study. However, the expert consensus at workshops held periodically to evaluate NHANES data collection procedures led to the decision to employ this method consistently in NHANES over the years, and this 24‐h recall is administered in person by trained dietary interviewers who are fluent in Spanish and English, which may make the 24‐h recall questionnaire as a viable method.

## 5 CONCLUSIONS

There was a negative association between dietary niacin intake and cognitive hypoacusis. Our results may be able to provide some reference for dietary prevention and treatment strategies for people with lower cognitive function, which has significant implications for the management of patients with cognitive impairment.

## AUTHOR CONTRIBUTIONS

**Xia Shen:** Conceptualization (equal); methodology (equal); resources (equal); software (equal); validation (equal); visualization (equal); writing – original draft (equal); writing – review and editing (equal). **Long Yang:** Data curation (equal); formal analysis (equal); software (equal); writing – original draft (equal); writing – review and editing (equal). **Yuan Yuan Liu:** Validation (equal); writing – original draft (equal); writing – review and editing (equal). **Lei Jiang:** Supervision (equal). **Jian Feng Huang:** Supervision (equal).

## CONFLICT OF INTEREST STATEMENT

The authors declare no conflict of interest.

## INSTITUTIONAL REVIEW BOARD STATEMENT

This study was supported by the National Center for Health Statistics Research Ethics Review Board, and the ethics approval number is Protocol #2011‐17 and Continuation of Protocol #2011‐17. You can find it at this website: NCHS Ethics Review Board Approval (cdc.gov).

## INFORMED CONSENT STATEMENT

This study is an analysis of the publicly available NHANES data. Informed consent was obtained from NHANES participants by the National Center for Health Statistics Research Ethics Review Board.

## Data Availability

All the data are available to the public and were used in the manuscript. The data can be available on the website: https://wwwn.cdc.gov/nchs/nhanes/search/default.aspx.
